# Mineral status and enteric methane production in dairy cows during different stages of lactation

**DOI:** 10.1186/s12917-021-02984-w

**Published:** 2021-08-28

**Authors:** Ľubomíra Grešáková, Monika Holodová, Małgorzata Szumacher-Strabel, Haihao Huang, Piotr Ślósarz, Janusz Wojtczak, Natalia Sowińska, Adam Cieślak

**Affiliations:** 1 Department of Digestive Tract Physiology, Institute of Animal Physiology, Centre of Biosciences of the Slovak Academy of Sciences, Šoltésovej 4, 040 01 Košice, Slovakia; 2 Department of Animal Nutrition, Poznań University of Life Sciences, Wołyńska 33, 60-637 Poznań, Poland; 3 Department of Animal Breeding and Animal Product Quality Assessment, Poznań University of Life Sciences, Słoneczna 1, 62-002 Złotniki, Poland; 4 Department of Genetics and Animal Breeding, Poznań University of Life Sciences, Wołyńska 33, 60-637 Poznań, Poland

**Keywords:** Holstein–Friesian cows, Macroelements, Trace elements, Methane emission, Lactation

## Abstract

**Background:**

Lactating dairy cows are the greatest livestock contributor of methane, a major global greenhouse gas (GHG). However, good feeding management with adequate mineral intake can offers an effective approach to maintaining high levels of milk production and the health of dairy cows over the entire course of lactation, while also helping to reduce methane emission. The study described here investigated the plasma concentrations of both macroelements (Ca, Na, K, Mg, P) and microelements (Zn, Cu, Fe, Mn), as well as enteric methane emission and milk composition in high-yielding dairy cows in different lactation periods. The experiment was performed on Holstein–Friesian dairy cows with the average milk yield of 41 (± 9) L/day in a Polish commercial farm with modern dairy systems. A total of thirty high-yielding dairy cows were randomly assigned into three groups differing by lactation stage: early stage (Early, days 25–100), middle stage (Middle, days 101–250), and late stage (Late, day 250 and later). Dietary treatment for all cows was a total mixture ration (TMR) with maize and alfalfa silage the main forage components.

**Results:**

The greatest milk yield and methane production were recorded in early-stage lactating cows, but the greatest methane intensity per kg of corrected milk was recorded in the late stage of lactation. Plasma concentrations of macroelements and microelements did not differ by lactation stages, but increased plasma concentrations of Zn and Fe and decreased plasma levels of Mg were noted during lactation. A positive correlation was found between plasma levels of Mg and other macroelements (Ca, Na, K), and between the concentrations of Fe and Zn, P in plasma, but no correlation between methane emission and mineral status was detected in the different lactation stages.

**Conclusions:**

Our results showed different mineral requirements and enteric methane emissions in each lactation stage. The feeding strategy and mineral utilization were adequate to maintain the health, mineral status, and milk production of the Holstein cows during the entire lactation period, and suggest an effective way of reducing methane emission.

## Background

The dairy sector is the greatest source of enteric methane contributing to agricultural greenhouse gas (GHG) emissions [1]. Enteric methane production is closely associated with the genetics, health, and productivity of dairy cows, as well as with feeding and nutrition management. Methane emission mitigation strategies in modern dairy systems include new technologies that assess CH_4_ concentrations, for example by measuring methane eructation during eating at robotic milking stations [1, 2]. Farmers try to mitigate GHG emissions and maintain the high productivity of dairy cows through genetic and feed-management approaches. New feeding strategies in the dairy sector, such as the replacement of grass silage with maize silage or increased feeding of concentrates in the total mixture ration (TMR), can alter rumen microbial activity so as to reduce methane output per volume of milk yield [3, 4]. Feed composition can affect the rumen microbe population responsible for rumen methane production, but the altered rumen environment may in turn affect nutrient utilization. Changes in rumen population—especially in rumen protozoa—can affect the metabolism and bioavailability of minerals; one example of this is the altered sulfur metabolism seen in microbial proteins, which secondarily affects the bioavailability of Cu and other trace minerals [5].

Milk production and lifetime performance play an important role in the breeding of high-production dairy cattle like Holstein cows. Sufficient mineral feed intake is thus necessary to maintain high levels of milk production, as well as the animals’ physiological and health status over the entire course of lactation. The milk production attendant on calving requires greater nutrient and mineral intake. High-yielding cows’ greater demand for the nutrients needed to synthesize milk can lead to negative energy balances and micronutrient deficiencies in the early stages of lactation [6]. Plasma mineral concentrations vary over the course of lactation, not only because of variations in mineral feed intake, but also as a result of the use of macroelements and microelements in metabolic processes [7, 8]. Monitoring the mineral status can provide valuable data on the nutrition and physiological status of dairy cows at particular lactation stages. However, published evidence on the association between mineral status and methane emissions is limited, so we hypothesize interactions between methane emission and mineral status at the early, middle, and late stages of lactation.

The main aim of our study was to monitor the enteric methane emission and mineral status of high-yielding dairy cows and to relate this to their lactation stages, in a modern farm system using a new feeding management strategy to mitigate CH_4_. Milk yield and milk chemical composition were also investigated in the different lactation periods and the correlations between measured data were evaluated using correlation analysis.

## Results

The chemical composition and mineral content of the TMR met the requirements of lactating dairy cows in each lactation stage (Table 1) [9]. Dry matter intake (DMI), nutrient, and mineral daily intake were estimated for each lactation stage (Table 2). The highest nutrient requirements and daily intake were observed during early lactation, and this decreased with lactation stage (LS).

**Table 1:** Ingredients, and chemical and mineral composition of a total mixed ration (TMR)

	Lactation stage^1^		
Item	Early	Middle	Late
Ingredients, g/kg of DM			
Maize silage	249	288	334
Alfalfa silage	198	229	266
Wet distillers’ grains	71	82	95
Brewery	43	50	59
Beet pulp	50	58	68
Carrot	10	12	13
Rape seed meal	61	70	81
Wheat meal	50	58	68
Mineral supplements	16	16	16
Commercial concentrate^2^	237	137	0
Energize^3^	15	0	0
Forage: concentrate ratio	62:38	72:28	84:16
Chemical composition,^4^ g/kg of DM			
OM	912	916	912
Ash	88	87	88
CP	179	185	188
EE	51	41	41
CF	162	180	200
aNDF	344	368	391
ADF	208	230	253
Macro mineral composition, g/kg DM			
Ca	8.1	7.1	7.1
Na	4.8	4.5	4.7
K	11.4	12.3	13.5
Mg	3.3	3.1	2.9
Micro mineral composition, mg/kg DM			
Fe	287	327	375
Mn	62	64	64
Zn	75	77	77
Cu	29	29	29

^1^Early = early-stage lactation (1–100 days); Middle = middle-stage lactation (101–250 days); Late = late-stage lactation (> 250 days)

^2^Stated to contain (as g/kg of DM in concentrate) OM (900), VEM (1021), aNDFom (250), CP (21.5), EE (29)

^3^Stated to contain (as g/kg of DM in concentrate) OM (980), VEM (2067), EE (850)

^4^Chemical composition: OM = organic matter; CP = crude protein; EE = ether extract; CF = crude fiber; aNDF = neutral detergent fiber; ADF = acid detergent fiber; VEM = net energy

**Table 2:** Daily intake of components and the minerals of dairy cows, by lactation state

	Lactation stage^1^		
Item	Early	Middle	Late
Daily intake^2^, kg/day			
DM	29.8	25.7	22.2
OM	27.1	23.6	20.2
Ash	2.6	2.2	1.9
CP	5.3	4.8	4.2
EE	1.5	1.1	0.9
CF	4.8	4.6	4.4
aNDF	10.2	9.5	8.7
ADF	6.2	5.9	5.6
Macro mineral intake, g/day			
Ca	241	183	157
Na	143	116	104
K	339	316	299
Mg	98.2	79.7	64.3
Micro mineral intake, mg/day			
Fe	8.53	8.41	8.31
Mn	1.85	1.63	1.42
Zn	2.24	1.97	1.71
Cu	0.86	0.75	0.64

^1^Early = early-stage lactation (1–100 days); Middle = middle-stage lactation (101–250 days); Late = late-stage lactation (> 250 days)

^2^DM = dry matter; OM = organic matter; CP = crude protein; EE = ether extract; CF = crude fiber; aNDF = neutral detergent fiber; ADF = acid detergent fiber

Milk yield and chemical composition differed across lactation stages (Table 3). The highest levels of milk production and lactose yield were recorded in the early stage of lactation (*P* < 0.0001). Increased yields of energy-corrected milk (ECM), milk protein, fat, and casein were noted in the early and middle LS (*P* < 0.001). On the other hand, milk protein and casein content were higher in late lactating cows than in cows in other lactation stages (P < 0.0001). Decreased dry matter (DM) content, urea concentration, and urea:protein ratio were noted in the milk of early lactating cows. Somatic cell counts in the milk tended to increase in cows in the late LS (*P* = 0.084). No significant differences were noted in ECM, the yield of milk fat, protein, or casein between early and middle lactation stages. Milk lactose and milk fat content did not vary across LS.

**Table 3:** Milk yield, milk chemical composition and gas emission of dairy cows, by lactation state

	Lactation stage^1^			Statistics	
Items	Early	Middle	Late	SEM	*P*-value
Yield					
Milk, kg/d	50.43^a^	42.35^b^	31.31^c^	1.789	< 0.0001
ECM,^2^ kg/d	45.69^a^	40.51^a^	31.65^b^	1.438	< 0.0001
Fat, g/d	1720^a^	1544^a^	1232^b^	57.10	< 0.001
Protein, g/d	1483^a^	1379^a^	1119^b^	43.90	< 0.001
Casein, g/d	1170^a^	1089^a^	883.5^b^	34.97	< 0.001
Lactose, g/d	2500^a^	2084^b^	1511^c^	91.36	< 0.0001
Milk composition					
Fat, %	3.425	3.665	3.931	0.5543	0.053
Protein, %	2.943^a^	3.271^b^	3.580^c^	0.0646	< 0.0001
Casein, %	2.322^a^	2.584^b^	2.824^c^	0.0526	< 0.0001
Lactose, %	4.958	9.924	4.819	0.0293	0.130
Dry matter, %	11.97^a^	12.48^ab^	12.96^b^	0.1228	< 0.01
Urea, mg/L	225.2^a^	297.8^b^	326.6^b^	10.06	< 0.0001
Somatic cells, 10^3^/mL	91.5	100.0	126.6	18.61	0.084
Urea/protein ratio	76.63^a^	91.32^b^	91.69^b^	2.339	0.007
Methane emission					
Methane production, ppm	606.9^a^	507.9^b^	471.2^b^	12.87	< 0.0001
Methane yield, ppm/kg DMI^3^	20.40^a^	16.87^b^	15.84^b^	0.441	< 0.0001
Methane intensity, ppm/kg ECM	13.45^ab^	12.47^a^	15.44^b^	0.457	0.020
CO_2_ production, ppm	5611^a^	4118^b^	4447^ab^	251.8	< 0.05

^a-c^Mean values in the same row with different superscripts are significantly different (P < 0.05), as determined by Tukey’s post-hoc test

^1^Early = early-stage lactation (1–100 days); Middle = middle-stage lactation (101–250 days); Late = late-stage lactation (> 250 days)

^2^ECM = energy-corrected milk

^3^DMI = dry matter intake

The significantly greatest level of production of methane and carbon dioxide (*P* < 0.0001, *P* < 0.05, respectively), as well as of methane yield per ppm of DMI (CH_4_/DMI, P < 0.0001), were seen in the early lactation stage. No significant differences in methane or carbon dioxide production or CH_4_:DMI ratio were noted between the middle and late LS. Methane intensity per kg of energy-corrected milk (CH_4_/ECM) was greatest in late-lactating cows (*P* < 0.05).

The plasma concentrations of macrominerals (Ca, K, Na, Mg, P) and microelements (Fe, Mn, Zn, Cu) did not differ by lactation stage (Table 4). Correlation analysis showed decreasing plasma concentrations of Mg with increasing number of days of lactation (*P* < 0.05), but a positive correlation was found between plasma concentrations of Mg and other macroelements (Ca, Na, K; all *P* < 0.05). A positive correlation was found between the day of lactation and plasma concentrations of Zn and Fe (*P* = 0.015, *P* < 0.05, respectively). Plasma Fe levels positively correlated with plasma concentrations of P (*P* < 0.001) and Zn (P < 0.05). Plasma albumin also positively correlated with Zn levels (P < 0.05), but negatively correlated with plasma K and Mg concentrations (P < 0.001, P < 0.05; respectively). Plasma Zn correlated with the count of somatic cells in the milk (*P* = 0.03) and plasma Cu levels correlated with milk protein and casein content (*P* < 0.01).

**Table 4:** Plasma concentrations of macroelements and microelements of dairy cows, by lactation states

	Lactation stage^1^			Statistics	
Minerals	Early	Middle	Late	SEM	P-value
Macroelements					
Na, g/L	3.12	3.08	3.12	0.013	0.311
K, mg/dL	16.3	15.8	16.1	0.230	0.689
P, mg/dL	5.95	5.86	5.94	0.129	0.954
Ca, mg/dL	8.04	8.32	8.69	0.173	0.316
Mg, mg/dL	2.43	2.25	2.30	0.035	0.095
Microelements					
Fe, mg/L	1.799	1.823	2.006	0.074	0.467
Zn, mg/L	0.774	0.777	0.885	0.025	0.105
Cu, mg/L	0.729	0.805	0.788	0.028	0.517
Mn, μg/L	14.56	13.20	15.58	1.557	0.842
Zn/Cu ratio	1.204	1.084	1.017	0.053	0.427
Albumin, g/L	33.68	36.51	36.85	1.465	0.607

^1^Early = early-stage lactation (1–100 days); Middle = middle-stage lactation (101–250 days); Late = late-stage lactation (> 250 days)

**Fig. 1:**
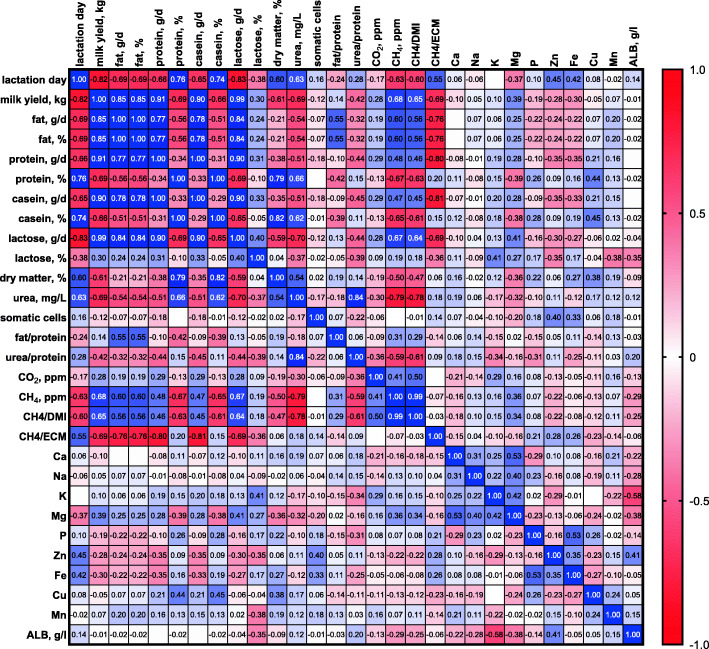
Pearson’s correlation coefficient for lactation day, milk chemical composition, and mineral plasma concentrations of dairy cows

Pearson’s correlation coefficient (r) was used to determine if any significant correlation could be found between the measured data (Fig. 1). Milk yield and milk composition, plasma concentrations of a few minerals, and methane emission data were affected by the day of lactation. An increasing number of days of lactation was strongly negatively correlated with milk yield and milk protein yield (both *P* < 0.0001), and positively correlated with milk protein and casein content (P < 0.0001), dry matter (*P* < 0.01) and urea milk content (*P* < 0.001), and milk fat yield (*P* < 0.05). A strong negative correlation was found between the lactation day and methane production and methane yield (P < 0.001, P < 0.0001, respectively). On the other hand, the day of lactation was positively correlated with methane intensity (CH_4_/ECM, *P* = 0.002).

## Discussion

Methane emissions vary across physiological stages and lifetime periods of dairy cows. In particular, they can be significantly affected by the amount of feed intake, the forage-to-concentrate ratio, the type of carbohydrate, forage preservation, and feeding frequency [1, 10]. Increased methane emission has been reported in dairy cows during the late stages of lactation, probably due to increased DMI from the forage components in this period [11, 12]. Even when DMI does not change, methane emission and yield differ across lactation stages, with the highest levels in late lactation [4, 11, 13]. We decided to show methane results as parts per million, even though most references for methane emission present their data as L or mL of methane concentration. We nevertheless believe that this may point to new directions of research based on farm monitoring systems that provide a low-cost and reliable method to estimate the daily methane output of individual dairy cows, which could be used to test the outcomes of mitigation strategies [2, 14]. In our study, on the basis of ppm concentration, we observed a decrease in the DMI of dairy cows and reduced milk yield with increasing lactation days; methane production followed a similar pattern, with a strong positive correlation between these values. Regardless of DMI, the highest methane yield was measured in cows in the early lactation stage. It seems that the increased production of methane at the beginning of lactation was caused by the high levels of milk synthesis and the mobilization of nutrients from somatic tissues, which may alter the energy balance of the lactating cows. Enteric methane emissions also increased due to increasing nutrient demand. On the other hand, the methane intensity expressed per kg of energy-corrected milk yield—which is the most useful and correct basis—was lower in middle lactation-period cows with higher milk production. Increased DMI, as well as increased concentrate feeding by dairy cows in the early and middle lactation stage, could have led to the decreased enteric methane emission per unit of ECM in our study. Feeding management approaches that aim to reduce methane emissions in dairy systems recommend increasing concentrate feeding and DMI or grain processing, altering rumen fermentation [1]. Including a greater proportion of concentrates and replacing grass silage by maize silage in TMR could reduce the CH_4_:ECM ratio by 2 to 6% for each kilogram DMI, or for every 1% increase in nonfiber carbohydrates in the daily ration, mainly because of the shift of NDF and starch digestion from the rumen to the small intestine [1, 3, 15]. We observed 1.7 and 5.5% decreases in CH_4_:ECM for each kilogram increase in DMI in early-stage and middle-stage lactating cows, respectively. The decrease in CH_4_/ECM ranged from about 0.5 to 1.4% for every 1% increase in concentrate intake in our study. It should also be underlined that, due to the very high quality of the forage used in the test diets, the total forage content (62%) in TMR was high, even in the early lactation group. Fermentation in the rumen, including methane production, depends to a large extent on the amount and type of fermented products consumed, so the use of a TMR with a high level of forage in the early lactation group enhances the methane production process, which was also observed in the present study.

The nutrient and mineral requirements for lactating dairy cows depend on their physiological stage, lactation period, milk production, and milk composition; adequate dietary mineral supplementation of high-yielding dairy cows is thus necessary to maintain their high productivity, reproductive ability, and health [16]. Macroelements are required by dairy cows for normal bone growth, reproductive performance, and milk production. Since mineral requirements vary during lactation, plasma mineral concentrations also fluctuate, due to differences in mineral feed intake, milk formation, metabolism, and mineral utilization in metabolic processes [7]. We recorded decreasing plasma Mg concentration with increasing days of lactation and a positive correlation between Mg and other macroelement concentrations in plasma, caused by both lower intake and decreased mineral absorption from the gastrointestinal tract during lactation. The most critical period in a productive dairy cow’s life is parturition and the beginning of lactation, when the nutritional and mineral demands for macroelements increase. Increased intake of Ca, P, and Mg in the early lactation stage is required to increase milk synthesis, and the absorption of these from the GIT increases as a result of homeostatic regulation mechanisms; serum concentrations of the macroelements are thus mainly affected by nutrition [17, 18]. A higher demand for Mg for milk synthesis was suggested by the positive correlation between plasma Mg levels and milk production seen in our study.

Trace minerals are crucial for proper immune response, the endocrine system, enzyme functions, and udder health [19]. Unlike serum macromineral concentrations which are mainly affected by nutrition, trace element concentrations in plasma (which do not remain stable during lactation) are independent of intake and are regulated by gut absorption and changing metabolic demands [20]. Although plasma concentrations of trace elements did not vary across the lactation stages in our study, an effect of days of lactation on plasma concentrations of Fe and Zn was noted. The increase in plasma Fe and Zn concentrations with days of lactation may relate to these minerals being increasingly required for milk synthesis in the early postpartum period, which often manifests as decreased plasma concentrations of Fe, Zn, and Cu [16, 21]. Zn is necessary for colostrum synthesis, and the increase in reactive oxygen species generated in the postpartum period stimulates the synthesis of Zn and Fe-dependent metalloproteins, resulting in reductions of these trace mineral plasma concentrations.

Our results do not allow us to confirm our hypothesis on the interaction between methane emission and mineral status in different lactation stages (early, middle, and late). However, there is some evidence that mineral supplementation reduces enteric methane emission by decreasing the density of methanogenic archaea related to lactation stages [22]. In any case, some studies have indicated that concentrations of milk minerals could be related to the lactation period of dairy cows [23, 24]. Further studies should thus also consider the mineral content of milk.

The beneficial effects of microelements (mainly Zn, Cu, and Se) on immunity, udder health, and milk composition have been demonstrated [25]. Despite their essential function in metal-containing enzymes—which can affect the antioxidant capacity and function of inflammatory cells—Cu can alter milks’ fatty acid profile and lipid metabolism, resulting in changes in milk fat content [26, 27]. Our study noted a positive correlation between plasma Cu and milk protein and casein content. However, the beneficial effect of feed supplementation with Cu and Zn on lactose and protein content in milk has already been described [28], and further research is needed to determine the effects of these trace elements on protein metabolism in dairy cows.

## Conclusions

High-yielding Holstein cows produced the highest milk yield, resulting in high enteric methane emission in the early lactation stage, but the late lactating cows were the greatest contributors to methane emission, taking their milk production into account (CH_4_ intensity expressed per kg of energy-corrected milk yield). To maintain high productivity while reducing enteric methane emission, it is necessary to provide for dairy cows’ nutrient and mineral requirements in each lactation stage involving new feeding management. No interactions were detected between methane emission and mineral status in different lactation stages. Our results have shown the different mineral requirement of cows in each lactation stage due to the mineral concentration variability in plasma during lactation. Plasma concentrations of macrominerals (Ca, K, Na, Mg, P) and microelements (Fe, Mn, Zn, Cu) did not differ between dairy cows in different lactation stages, so we can state that the TMR composition was adequate for maintaining health, mineral status, and milk production of the Holstein cows in every lactation stage. Further study of the dynamics of macroelement and microelement metabolism during lactation is necessary for early diagnostics of alterations in nutritional and health status in high-yielding dairy cows.

## Methods

### Animals and diets

This study complied with the ARRIVE guidelines for animal research [29]. The experiment was performed at a commercial farm in Poland. All experimental procedures were carried out in accordance with the approval of the Local Ethical Commission for Investigations on Animals and was in line with Polish law.

A total of thirty multiparous high-yielding dairy cows (657 ± 17 kg of BW, milk yield 40 ± 9 L/d, average annual milk yield 12,000 L/cow) were randomly selected from a Polish Holstein–Friesian herd containing 120 lactating dairy cows on a commercial farm. For the thirty days prior to the experiment, the cows were fed the same diet served as a partial mixed ration (PMR) with appropriate amounts of concentrate, followed by nutrient requirements based on the milk yield. The PMR was formulated using FeedExpert software (Rovecom, Hoogeveen, the Netherlands). On day 31 of the experiment, thirty cows were selected by lactation stage: early-stage lactation (Early, days 25–100), middle-stage lactation (Middle, days 101–250), and late-stage lactation (Late, day 250 and later) and randomly allocated into three groups of ten animals each. The sample size was selected to find a difference in milk yield, with an alpha level of 0.05 and a power of 0.8 (GraphPad StatMate 2.00). A total of ten dairy cows per group (*n* = 10) were necessary for this, so thirty animals were used in the study. The cows from each group were kept separately in free open-stall housing with free access to water and an automated milking system (AMS; Lely Astronaut A5, Lely Industries, Maassluis, the Netherlands). Due to the limitations of running the experiment under production conditions, the cows were supervised by two workers for 24 h, who ensured that the cows returned from the AMS to the appropriate group (early, middle, and late lactation). The cows presented themselves for milking as in the period prior to the collection period (from two to six times a day, average 3.4 ± 0.89). The collection period of the experiment lasted eight days, from days 31 to 39. During this period, an appropriate total mix ration (TMR) was offered to all cows based on milk yield. TMRs were provided twice a day. The ingredients and chemical and mineral composition of TMR differing in the lactation stage are presented in Table 1.

The forage-to-concentrate ratio was altered for each lactation stage (62:38, 72:28, and 84:16, respectively, for the early, middle, and late lactation groups). Maize and alfalfa silage were used as the main forage components. The average feed intake was monitored daily for eight days for each group by weighing the total amounts offered and leftovers. Total daily amounts of TMR were divided by the number of cows in the group to calculate individual dry matter intake. Other nutrient components and mineral content were determined for each treatment using dry matter intake (Table 2). Gas production during milking at the AMS was continuously measured using an infrared methane analyzer (Servomex 4000 Series, Servomex, Jarvis Brook, UK), as previously described by Sypniewski et al. [2]. Additionally, milk yield and body weight were recorded, and milk samples were collected. The chemical composition of the milk was analyzed using an infrared analyzer (Milko-Scan 255 A/S N). Blood samples were collected from each cow after morning milking, during routine veterinary monitoring procedures on the farm. The milk and blood were sampled at the same day, together with methane measurements. Blood samples were taken from *v. abdominalis superficialis* into heparinized tubes and were centrifuged at 3000 *g* for 10 min. All plasma samples were stored at − 20 °C for further analysis. Feed samples were collected three times during the collection period.

### Analytical methods

The TMR was chemically analyzed using the procedures of AOAC [30] for DM (method no. 934.01), crude protein (CP; using a Kjel-Foss Automatic 16,210 analyzer; method no. 976.05), ash (method no. 942.05), crude fiber (CF; using FOSSTecator, Fibertec System, method 962.09), and ether extract (EE; using a Soxhlet System HT analyzer; method no. 973.18). Organic matter content was calculated by subtracting the ash concentration from DM content. The nitrogen-free extract was estimated by deducting the concentrations of crude fiber, CP, EE, and ash from the DM content.

Daily milk production and the chemical composition of the milk were quantified using an infrared analyzer (Milko-Scan 255 A/S N; Foss Electric, Hillerød, Denmark). The urea concentration of the milk was determined by infrared spectrometry using a Combi Foss 6000 analyzer (Foss Electric). The energy-corrected milk yield (ECM) for milk protein and fat content was calculated according to the following equation from van Lingen et al. [31]:

ECM (g/kg) = milk yield (kg/d) × (0.337 + 0.116 × milk fat (%) + 0.06 × milk protein (%)).

Methane and CO_2_ production were measured using infrared methane analyzers (Servomex 4000 Series, Servomex, Jarvis Brook, UK). The methane emission data include measured methane production (CH_4,_ ppm), methane yield per kg of dry matter intake (CH_4_/DMI, ppm/kg), and methane intensity per kg of energy-corrected milk (CH_4_/ECM, ppm/kg). The measurement of methane has been described in detail by Sypniewski et al. [2]. Briefly, an infrared methane analyzer was used in the AMS. Air samples were continuously collected using a gas panel. The gas samples were distributed to the inlet port of the analyzer with a flow rate of 4 L/min. Methane concentrations were measured at two-second intervals and the data were stored on a computer using software with a database system (RS 232; AnaGaz, Wrocław, Poland). Before measuring the methane concentrations of the gas samples, the analyzers were calibrated using a standard calibration gas (Multax, Zielonki-Parcela, Poland) containing 1210 ppm of methane in nitrogen gas (99.99%). MATLAB was used to identify and quantify peaks. Peaks with a height of less than 85 ppm were discarded; 85 ppm was taken as a baseline in this barn.

The mineral content of the TMR was analyzed using flame atomic absorption spectrophotometry with a double-beam atomic absorption spectrophotometer (AA-7000 Series, Shimadzu Co., Kyoto, Japan) in six replicates. The certificate reference materials of Feed LGC7173, (LGC Standards, UK) were included in each analysis to verify the accuracy of the instrument.

Plasma concentrations of Ca, Na, K, Mg, and microelements Zn, Cu, Fe were analyzed by flame atomic absorption spectrophotometry using the atomic absorption spectrophotometer (AA-7000 Series, Shimadzu Co., Kyoto, Japan). Plasma phosphorus was determined using commercial diagnostic kits (Randox, UK) on an automatic biochemical analyzer (Alizé, Lisabio, France). The direct determination of plasma Mn content was analyzed by atomic absorption spectrophotometry with electrothermal atomization (ETAAS) using the AAS with a graphite furnace (GFA-7000, Shimadzu Co., Kyoto, Japan) via the SR (high-speed self-reversal) method of background correction and pyrolytic-coated graphite tubes. The certificate reference material of lyophilized human plasma ClinCheck Control (Recipe, Munich, Germany) was included in each analysis to verify the accuracy of the instrument.

Plasma albumin content was evaluated photometrically using an ALB500 commercial kit (Erba Lachema, Czech Republic).

### Statistical analysis

No animals, experimental units, or data points were excluded from the statistical analysis. The data was statistically analyzed using GraphPad Prism statistical software (version 9.0.0, GraphPad Software, San Diego, CA, USA). For multiple comparisons, one-way analysis of variance (ANOVA) was used, followed by a Tukey’s post-hoc test. Pearson’s correlation analysis was carried out between the plasma macroelement and microelement concentrations, milk chemical compositions, and methane data, to determine the relationships between the data and the strength of the putative linear association between the variables. The differences between the mean values of the lactation groups were considered to be statistically significant at *P* < 0.05. The values in the tables are means and pooled standard errors of the mean (SEM).

## Data Availability

The datasets used and analyzed in this survey are available from the corresponding authors upon reasonable request.

## Abbreviations

AMS: automated milking system
AOAC: Association of Official Analytical Chemists
CF: crude fiber
CH_4_/DMI: Methane yield per ppm of DMI
CH_4_/ECM: Methane intensity per kg of energy-corrected milk
CP: Crude protein
DM: Dry matter
DMI: Dry matter intake
ECM: Energy-corrected milk
EE: Ether extract
GHG: Global greenhouse gas
GIT: Gastrointestinal tract
LS: Lactation stage
PMR: Partial mixed ration
TMR: Total mixture ration
